# Feasibility and outcomes of break-upward side-lying positioning after PPV for inferior rhegmatogenous retinal detachment: a retrospective case series

**DOI:** 10.3389/fmed.2026.1865334

**Published:** 2026-06-26

**Authors:** Huilan Zeng, Shuyan Yang, Shangyu Yang, Zishan Yang, Jianming Sun, Qiuping Liu

**Affiliations:** 1 Department of Ophthalmology, The First Affiliated Hospital, Hengyang Medical School, University of South China, Hengyang, Hunan, China; 2 University of South China, Hengyang, Hunan, China

**Keywords:** inferior retinal breaks, pars plana vitrectomy, postoperative positioning, rhegmatogenous retinal detachment, side-lying position

## Abstract

**Objective:**

To evaluate the feasibility and clinical outcomes of adopting a break-upward side-lying position after silicone oil tamponade in patients with inferior rhegmatogenous retinal detachment.

**Methods:**

A retrospective analysis was conducted on 24 patients (24 eyes) with inferior rhegmatogenous retinal detachment who underwent pars plana vitrectomy (PPV) combined with silicone oil tamponade at our hospital between January 2023 and December 2024. All patients adopted a break-upward side-lying position postoperatively and were followed for at least 6 months. Outcome measures included retinal reattachment rate, break closure, changes in best-corrected visual acuity (BCVA), incidence of complications, and patient adherence to postoperative positioning.

**Results:**

All 24 patients achieved complete retinal reattachment with full closure of the retinal breaks, yielding a 100% re-attachment rate. BCVA showed varying degrees of improvement at 6 months postoperatively. Only two patients developed elevated intraocular pressure, which resolved with medical treatment. No significant anterior chamber inflammation, corneal complications, or silicone oil migration was observed. Patient adherence and comfort with the prescribed positioning were rated as good. Our findings suggest that side-lying positioning strategy is a feasible and safe postoperative management approach for patients undergoing PPV with silicone oil tamponade for inferior rhegmatogenous retinal detachment.

**Conclusion:**

In patients with inferior rhegmatogenous retinal detachment treated with silicone oil tamponade, break-upward side-lying positioning is a safe, effective, and well-tolerated postoperative management strategy, and may serve as a valuable supplement or alternative to the traditional prone position.

## Introduction

Rhegmatogenous retinal detachment (RRD) is a common vision-threatening ophthalmic disease. Without timely treatment, patients are likely to experience irreversible and permanent loss of visual function (1, 2). With the advancement of minimally invasive PPV, combined tamponade with silicone oil or gas has become an essential approach for the treatment of retinal detachment (3). As an intraocular tamponade, silicone oil offers unique advantages in managing retinal re-attachment due to its relatively low specific gravity and stable physical properties (4). However, the physical properties of silicone oil cause it to float in the upper part of the vitreous cavity, creating an oil–fluid interface. To ensure coverage of *inferior retinal breaks* by the silicone oil, patients are typically instructed to maintain a prone position postoperatively (5, 6). Although theoretically reasonable, prolonged prone positioning can significantly affect patients’ quality of life in clinical practice. Common issues include elevated intraocular pressure, respiratory difficulties, neck and back pain, sleep disturbances, and psychological stress (7–10), which may prevent some patients from strictly adhering to medical instructions and thus adversely affect treatment outcomes. In recent years, the concept of “individualized postoperative positioning” has gradually emerged. In the surgical management of conditions such as macular holes or epiretinal membranes, randomized controlled trials have demonstrated that strict prone positioning is not always necessary, and some patients can achieve favorable outcomes with supine or side-lying positions (11, 12). However, clinical evidence on the use of a *break-upward side-lying position* in patients with silicone oil–tamponade inferior rhegmatogenous retinal detachment remains very limited. In this study, we retrospectively analyzed 24 patients with inferior retinal breaks who all adopted a break-upward side-lying position after silicone oil tamponade. Our aim was to evaluate whether this positioning could maintain anatomical efficacy while improving patient adherence. Interpreted in light of previously reported postoperative positioning strategies, our findings may provide additional evidence to support individualized postoperative positioning recommendations in clinical practice.

## Methods

### Study design and ethics

This study was a single-center, retrospective case series. The study protocol was approved by the Ethics Committee of The First Affiliated Hospital of the University of South China.

Demographic and clinical data, including sex, age, and self-reported symptom duration, were obtained from the inpatient medical record system. Lens status was assessed according to the Lens Opacities Classification System (LOCS) based on slit-lamp examination records. The number, location, and size of retinal breaks were extracted from preoperative fundus examination records. The extent of retinal detachment and the severity of proliferative vitreoretinopathy (PVR) were evaluated using ultra-widefield fundus imaging. PVR was graded according to the Retina Society Classification of Proliferative Vitreoretinopathy (1983). Macular involvement was determined based on optical coherence tomography (OCT) findings. Best-corrected visual acuity (BCVA) was converted to logarithm of the minimum angle of resolution (LogMAR) units for statistical analysis. Follow-up data were collected from outpatient medical records.

### Patient data

*Inclusion criteria*: (1) RRD; (2) retinal breaks located between the 4 o’clock and 8 o’clock positions; (3) first-time treatment with PPV combined with silicone oil tamponade; (4) follow-up duration ≥6 months.

*Exclusion criteria*: (1) choroidal detachment; (2) diabetic retinopathy or severe concomitant fundus disorders; (3) history of prior intraocular surgery.

A total of 24 patients (24 eyes) were ultimately included in the study.

### Surgical procedure

All surgeries were performed by the same surgeon. A 25-gauge PPV system was used. Under a non-contact wide-angle viewing system, the central and posterior vitreous was removed, followed by peripheral vitreous removal under scleral depression. Previous studies have reported that intraoperative Perfluorocarbon Liquid (PFCL) use may be associated with an increased risk of postoperative ellipsoid zone/interdigitation zone (EZ/IZ) disruption (13). Moreover, PFCL is not essential for retinal reattachment in many cases of RRD (14). Therefore, fluid–air exchange was performed without PFCL. Subretinal fluid was aspirated as completely as possible using suction to facilitate retinal reattachment. Retinal breaks were sealed with laser photocoagulation. At the completion of surgery, the vitreous cavity was completely filled with silicone oil, with the injected volume adjusted according to the size of the vitreous cavity and intraoperative conditions.

### Postoperative positioning

**Figure 1:**
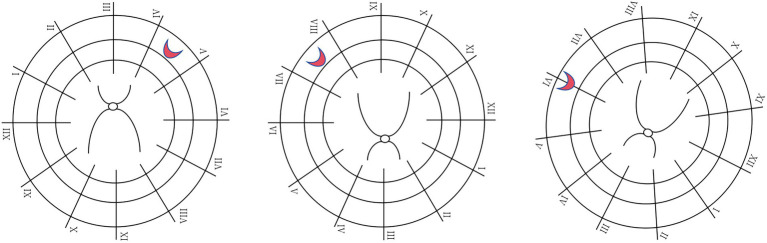
Schematic illustration of the break-upward positioning strategy for inferior retinal breaks at different locations in the right eye.

All patients were instructed to adopt a break-upward side-lying position immediately after surgery, maintaining it for more than 16 h per day for 4 weeks, including during the night. The nursing team provided patients with training and guidance on proper positioning. The positioning strategy was individualized according to the location of the retinal break, with the primary objective of keeping the break upward relative to gravity. For retinal breaks located near the 6 o’clock meridian, patients were instructed to adopt a contralateral side-lying position without a pillow. The operated eye was positioned uppermost, and the resulting slight elevation of the chin and mild neck extension allowed the inferior retinal break to be oriented above the lowest gravitational point, thereby maximizing silicone oil tamponade over the break (Figure 1).

### Outcome measures

Patients were assessed at 1 week, 1 month, and 6 months postoperatively. The outcome measures included: (1) retinal reattachment rate and closure of retinal breaks; (2) changes in BCVA from pre- to post-surgery; and (3) incidence of complications.

## Results

A total of 24 patients met the inclusion criteria and did not meet any exclusion criteria (Table 1), including 13 men and 11 women. The mean age was 48.7 ± 12.6 years. The duration of disease ranged from 1 day to 3 months, and the size of the retinal breaks ranged from 0.5 to 4-Papilla Diameter (PD). Nineteen patients had macular involvement, while 5 patients did not. Although the self-reported duration of symptoms was relatively short in some patients, fundus examination often demonstrated advanced PVR changes. This finding suggests that retinal detachments caused by inferior retinal breaks may progress insidiously, as subretinal fluid tends to accumulate gradually in the inferior retina and may initially spare the macula. Consequently, patients may remain relatively asymptomatic and underestimate the true duration of retinal detachment.

**Table 1:** Baseline characteristics of patients.

NO.	Gender	Age	Hx	LOCS	Location of the breaks	Size of the breaks	Area of the retinal detachment	Macular involvement	PVR	Pre-surgery LogMAR BCVA
1	Female	57	1 M	C3N2P1	6-o’clock, 2PD posterior to the ora serrata	Gravel-like Microholes	40%	Yes	C2	1
2	Female	58	10 + D	CtrN1P0	7-o’clock	3PD	45%	Yes	C2	0.82
3	Male	42	10 + D	CON1P0	6-o’clock	4PD	70%	Yes	C1	1.7
4	Female	45	2 W	C0N0P0	5-o’clock	1PD	60%	Yes	C2	1.9
5	Male	19	1 W	C0NOPO	7-o’clock	3PD × 2	75%	Yes	D1	2.3
6	Male	39	1 W	C0NOP0	7–8-o’clock	Cobblestone	40%	Yes	B	0.6
7	Female	61	20 + D	C1N1P0	7-o’clock	2PD × 2	40%	No	C1	0.1
8	Male	41	1D	C0N0P0	6-o’clock	1PD × 2	80%	Yes	C3	1.9
9	Female	42	3D	CON1P0	7-o’clock	2PD	40%	Yes	B	1
10	Male	44	1 W	C1N1P0	8-o’clock	Multiple small holes	40%	Yes	B	0.7
11	Female	61	20 + D	C1N2P1	5-o’clock	0.5PD	60%	Yes	C3	1.7
12	Female	60	10 + D	C2N3P1	5-o’clock	1.5PD	70%	Yes	C3	1.4
13	Male	46	10 + D	C1N1P0	5–7-o’clock	0.5PD, 2PD	70%	No	C3	0.7
14	Male	43	1 M	C0N1P0	7-o’clock	0.5PD	20%	No	A	1.22
15	Male	66	10 + D	CtrN1P0	7-o’clock	2PD	70%	Yes	C1	1.7
16	Male	15	2 M	CON1P0	7-o’clock	2PD	50%	Yes	B	2
17	Male	52	1 M	C1N1P0	6-o’clock	4PD	80%	Yes	C1	2.3
18	Female	59	1 W	C1N1P1	8-o’clock	1PD	60%	No	C2	1.1
19	Male	52	3 M	C0N1P0	7–8-o’clock	5PD, 0.5PD	70%	Yes	C1	1.1
20	Female	56	1 M	C0N0P0	5-o’clock	0.5PD	70%	Yes	C2	0.92
21	Female	62	1 W	C1N1P0	7-o’clock	1.5PD	80%	Yes	D1	2.3
22	Male	55	3D	CtrN0P0	6-o’clock	3PD	40%	No	C2	0.22
23	Female	48	1 W	CtrN2P0	5–6-o’clock	Gravel-like microholes	50%	Yes	C1	0.7
24	Male	45	5D	C0N0P0	3–6-o’clock	4PD	80%	Yes	C3	2.3

Variables: sex, age, duration of disease, LOCS, retinal break location, break size, extent of retinal detachment, macular involvement, PVR and preoperative BCVA in LogMAR.

At the 6-month follow-up, silicone oil removal had been performed in 18 patients, while 6 patients remained under silicone oil tamponade. Of the 18 patients who underwent silicone oil removal, 15 underwent combined phacoemulsification and intraocular lens implantation at the time of oil removal, whereas the other 3 underwent silicone oil removal without concurrent cataract surgery.

### Anatomical outcomes

**Figure 2:**
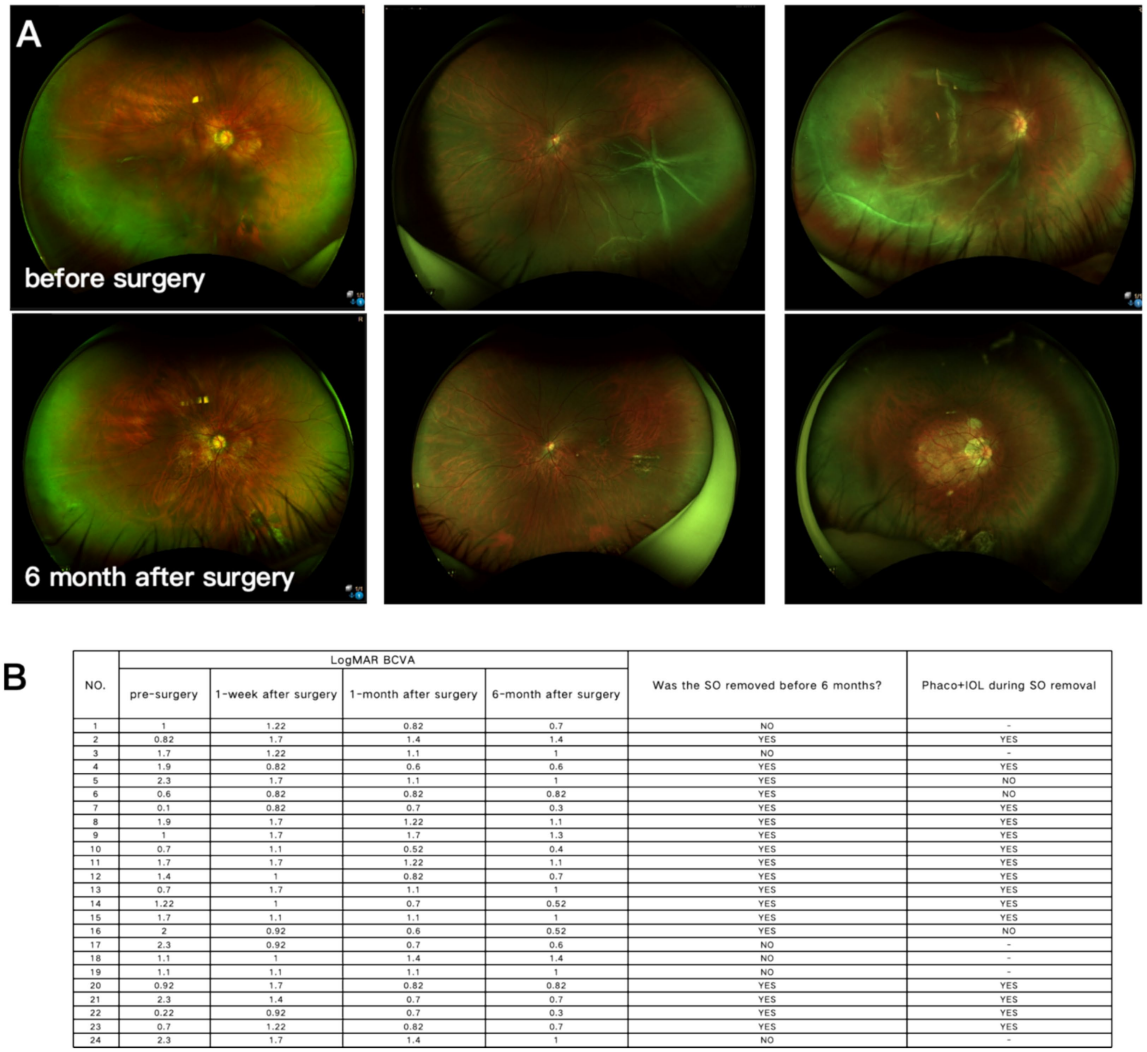
Anatomical reattachment and visual acuity changes before and after surgery. **(A)** Comparison of retinal photographs before and after surgery. **(B)** Preoperative and postoperative LogMAR BCVA of individual patients.

All 24 patients achieved complete retinal reattachment with full closure of retinal breaks at the 6-month. Among the 18 eyes that underwent silicone oil removal during follow-up, retinal attachment was maintained in all cases after oil removal (Figure 2A).

### Functional outcomes

Considering that visual acuity at postoperative week 1 may be affected by transient factors such as corneal edema, anterior chamber inflammation, pharmacologic mydriasis, and intraocular pressure fluctuations, whereas visual outcomes at 6 months may be influenced by differences in silicone oil removal status and concomitant cataract surgery, postoperative month 1 BCVA was selected as the primary visual outcome measure.

All visual acuity values were converted to logarithm of the minimum angle of resolution (LogMAR) units for statistical analysis. Data are presented as mean ± standard deviation (SD). Statistical analyses were performed using GraphPad Prism 9 (GraphPad Software, San Diego, CA, USA). The normality of the paired differences between preoperative and postoperative month 1 BCVA was assessed using the Shapiro–Wilk test and was found to be normally distributed (W = 0.946, *p* = 0.225). Therefore, a paired t-test was used for comparison.

The mean preoperative BCVA was 1.320 ± 0.676 LogMAR, which improved to 0.965 ± 0.313 LogMAR at postoperative month 1. The mean improvement in BCVA was 0.355 LogMAR (95% CI, 0.052–0.658). Paired *t*-test analysis demonstrated a statistically significant improvement in BCVA at postoperative month 1 compared with baseline (*t* = 2.426, *p* = 0.024). The effect size was moderate (Cohen’s d = 0.495), indicating a clinically meaningful improvement in visual function following surgery (Figure 2B).

At the 6-month (BCVA was 0.833 ± 0.316 LogMAR), six patients exhibited BCVA below their preoperative level, whereas BCVA was maintained at or improved beyond baseline in the remaining 18 patients. The limited visual recovery observed in these patients may be related to silicone oil emulsification, cataract progression, posterior capsular opacification, or irreversible macular damage associated with retinal detachment (Figure 2B).

### Complications

Two patients developed elevated intraocular pressure, which normalized with medical treatment. One patient experienced mild anterior chamber inflammation, which resolved with topical corticosteroid drops. No corneal complications or silicone oil migration were observed.

### Adherence and comfort

Patients generally reported that the side-lying position was significantly more comfortable than the prone position, with no respiratory difficulties or other discomfort. No patient discontinued the prescribed positioning due to intolerance.

## Discussion

Most ophthalmic surgeons worldwide believe that postoperative positioning is critical to the success of pars plana vitrectomy with silicone oil tamponade for retinal detachment, with the first 24 h after surgery being particularly crucial. Previous studies have indicated that after silicone oil tamponade, patients need to maintain a face-down position for more than 16 h per day during the first month, after which the required duration can be gradually reduced depending on retinal recovery, until silicone oil removal (15). Other studies have shown that for patients with rhegmatogenous retinal detachment undergoing vitrectomy with silicone oil tamponade, maintaining a prone position immediately after surgery can help reduce retinal displacement (16, 17). However, inferior-break rhegmatogenous retinal detachment has long been a challenge in retinal reattachment surgery. Previous studies have reported that pars plana vitrectomy yields a success rate of only 76.8–95.4% for inferior retinal breaks (18–26). To improve the retinal reattachment rate, some investigators have adopted pars plana vitrectomy combined with scleral buckling for the treatment of inferior-break rhegmatogenous retinal detachment. However, this approach increases the risk of complications associated with scleral buckling (27), such as subchoroidal hemorrhage. Some retrospective analyses have suggested that combining pars plana vitrectomy with scleral buckling does not necessarily improve the surgical success rate (28–30). For inferior retinal breaks, some surgeons have even attempted postoperative head-down positioning (24), inverted positioning (31, 32), or leaving perfluoro-octane in the vitreous cavity followed by a second-stage vitrectomy (33). However, both prone and inverted positioning can cause considerable discomfort—including conjunctival edema (34), periorbital swelling (35), ischemic optic neuropathy (36), and even mesenteric thrombosis (37)—which substantially reduces patient adherence (9).

Therefore, to improve the surgical success rate for inferior-break rhegmatogenous retinal detachment and to minimize the complications associated with prone positioning, this study proposes a postoperative break-upward side-lying positioning strategy after vitrectomy. This approach achieved a 100% anatomical success rate with good patient tolerance in our cohort. The following discussion systematically addresses its theoretical basis from the perspectives of physics and fluid dynamics, patient adherence, comparison with domestic and international studies, and postoperative complications and their relationship to positioning.

### Physical and fluid dynamics principles of silicone oil

**Figure 3:**
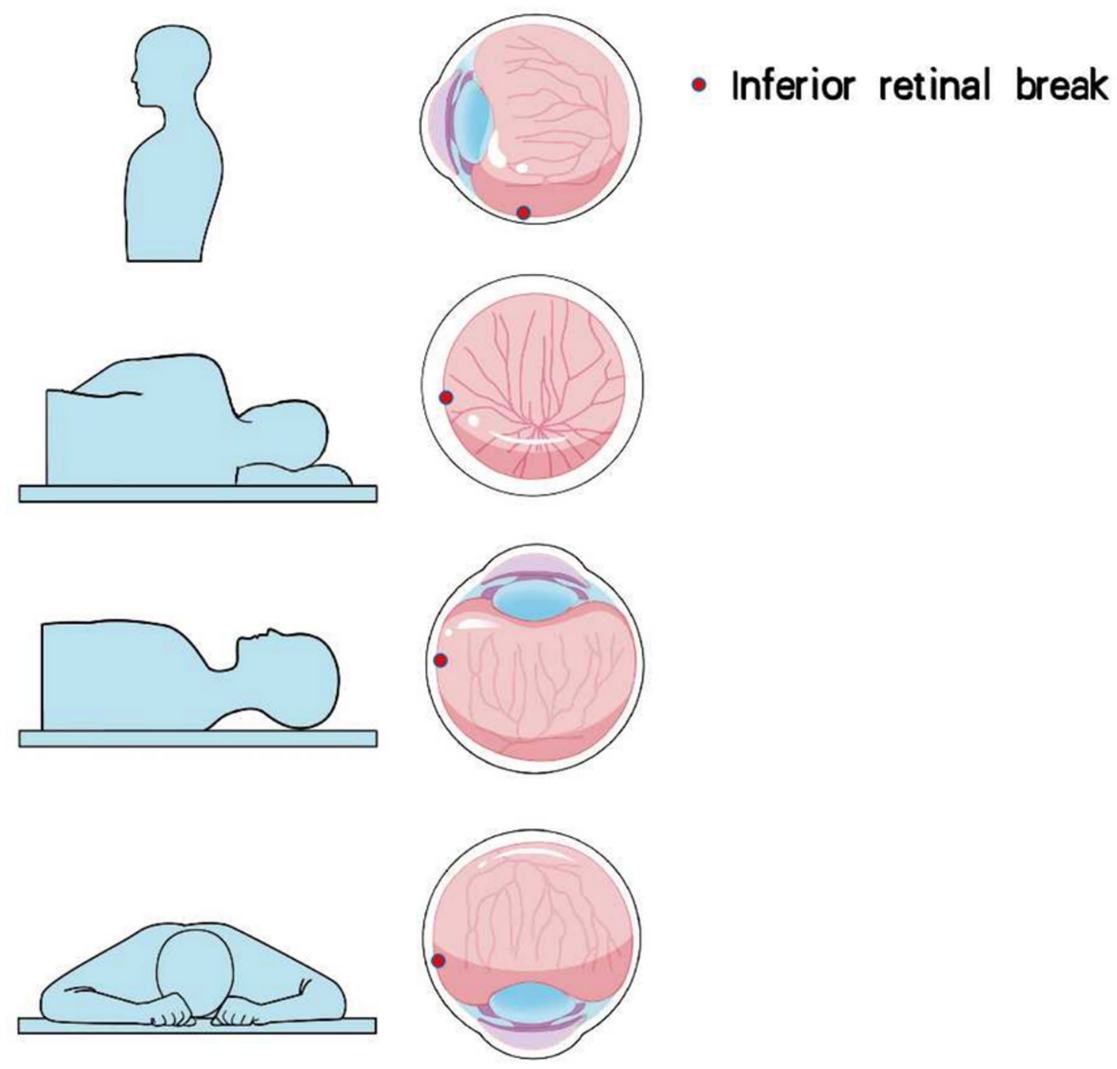
Schematic illustration of intraocular silicone oil distribution in different positions. Compared with the prone position, the side-lying position allows better coverage of inferior retinal breaks by intraocular silicone oil.

In retinal reattachment, the most critical steps are relieving vitreoretinal traction and achieving complete closure of retinal breaks (2). As an intraocular tamponade, silicone oil primarily relies on its low specific gravity and high surface tension to form a stable oil–fluid interface within the vitreous cavity, providing “interfacial pressure” and a “fluid barrier” over the retinal break. Classical views suggest that patients with inferior retinal breaks must maintain a prone position postoperatively to ensure adequate coverage by the silicone oil (15, 38–40). However, it should be noted that break closure and chorioretinal adhesion are more influenced by the surface tension of the tamponade rather than its buoyancy (41, 42). Moreover, the spatial distribution of intraocular silicone oil is not a static horizontal plane; it is a dynamic interface affected by gravity, globe geometry, the relative volumes of the anterior and posterior segments, and minor positional changes (16, 43). MRI studies and numerical simulations indicate that the oil–fluid interface forms a curved surface under different positions, and when the break is positioned “upward,” sufficient coverage can be achieved even without strict prone positioning (43). This provides a physical basis for the feasibility of the break-upward side-lying position. Therefore, we hypothesize that once the retinal breaks are sealed with laser photocoagulation, as long as the silicone oil interface covers the breaks, its surface tension can keep them dry and prevent fluid from entering the subretinal space. Compared with the prone position, a side-lying position may better ensure coverage of inferior retinal breaks by the silicone oil interface (Figure 3). Our observations indicate that for inferior retinal breaks, postoperative side-lying positioning resulted in a retinal reattachment rate higher than the levels generally reported in the literature.

### Limitations of the traditional prone position and issues with patient compliance

Prolonged prone positioning commonly leads to restricted respiration, upper-airway compression, gastroesophageal reflux, fatigue and pain in the cervical–lumbar–paraspinal muscle groups, as well as sleep disturbance and anxiety- or depression-like symptoms (15, 40). These risks and discomforts are even more pronounced in elderly or obese individuals and in patients with COPD or obstructive sleep apnea, often resulting in poor adherence to medical instructions. From an evidence-based perspective, the “superiority” of strict face-down positioning is not as strong as traditionally portrayed in textbooks. Randomized controlled trials have shown that position-of-break–oriented strategies are not inferior to a uniform face-down regimen for certain outcomes (15). The 2024 Cochrane review likewise indicates that evidence supporting the absolute necessity of prone positioning remains limited, and patient-specific pathology should guide flexible decision-making (40).

Poor compliance can compromise outcomes through two pathways: inadequate coverage time of the retinal break allows small amounts of fluid to re-enter the subretinal space, and frequent positional changes create fluctuations at the oil–fluid interface, leading to intermittent “break exposure.” In contrast, the lateral decubitus position aligns better with natural resting posture, substantially reduces respiratory burden and musculoskeletal stress, and enables patients to maintain the prescribed position more comfortably and sustainably. Thus, a positioning strategy that balances tamponade coverage and tolerability may more effectively promote retinal reattachment (11, 38, 44).

In our case series, all 24 eyes achieved durable reattachment throughout follow-up, with no interruptions due to position intolerance. This observation is consistent with prior investigations on “non-strict prone” or “supine/lateral alternative” strategies, which likewise report high anatomic success rates in patients with inferior-quadrant breaks or specific break distributions (11, 20, 22, 38, 44–46).

### Comparison and systematic analysis of domestic and international studies

An increasing number of vitreoretinal surgeons have been exploring ways to improve postoperative positioning. The earliest focus was on macular hole retinal detachment. As early as 1997, researchers proposed that prone positioning was not required: pars plana vitrectomy combined with intraocular C3F8 gas tamponade could effectively treat macular hole retinal detachment and achieve hole closure (47). For chronic idiopathic macular holes, vitrectomy with non–face-down positioning can achieve an anatomical closure rate of up to 94.4% (48). For macular holes smaller than 400 μm, prone positioning offers no clear advantage over other positions, whereas for holes larger than 400 μm, face-down positioning may increase the closure rate (49).

More recently, studies have indicated that, for rhegmatogenous retinal detachment other than macular hole–associated cases, prone positioning after vitrectomy provides no significant advantage over adjustable positioning in terms of anatomical reattachment or best-corrected visual acuity (12). For inferior retinal breaks, alternating supine and lateral positioning for 14 days after vitrectomy with gas tamponade has been shown to effectively treat primary rhegmatogenous retinal detachment involving the 4 to 8 o’clock meridians (50). In the clinical trial by Shiraki et al. (11), in patients with inferior retinal breaks who received 20% SF6 gas tamponade after PPV, the initial reattachment rate was significantly higher in the non-prone group than in the prone group, while no significant differences were observed between the two positions in patients without inferior breaks.

Taken together, these findings point to a shared conclusion: postoperative positioning should be strategically tailored to the location of the retinal breaks rather than relying on the prolonged maintenance of a fixed pose. Our results further support this approach from another perspective, demonstrating that patients can achieve high adherence while obtaining favorable anatomical outcomes.

### The relationship between silicone oil–related complications and postoperative positioning

Silicone oil–related complications include postoperative intraocular pressure (IOP) elevation/glaucoma, emulsification and anterior migration, corneal endothelial damage, inflammatory responses, and the risk of re-detachment after oil removal (51–58). Traditionally, it has been suggested that the absence of a face-down position after PPV with silicone oil tamponade may predispose patients to pupillary block glaucoma (PBG) (59), which is generally considered to be associated with a lax lens–iris diaphragm (60, 61). However, in patients with rhegmatogenous retinal detachment caused by inferior retinal breaks, PBG has not been observed even when a supine position was adopted after PPV (38). Moreover, in a study by Al-Jazzaf et al. (62) evaluating the incidence and characteristics of glaucoma in 450 eyes following vitrectomy with silicone oil injection, the authors reported that anterior migration of silicone oil does not necessarily lead to PBG.

Nevertheless, it is important to note that silicone oil tends to accumulate anteriorly when patients are in a supine position, posing risks of anterior migration and silicone oil herniation into the anterior chamber (63, 64); therefore, a supine position is not recommended after PPV with silicone oil tamponade.

In the present study, transient IOP elevation occurred in two eyes and resolved promptly with topical IOP-lowering medications. A potential concern is that improper execution of the lateral decubitus position may lead to intermittent exposure of the retinal break, theoretically allowing small amounts of fluid to re-enter the subretinal space. Thus, patient education and monitoring to ensure that the “retinal break remains continuously uppermost” are essential in clinical practice.

The limitations of this study include a small sample size, single-center design, retrospective nature, and the absence of a parallel control group. In addition, patient compliance with the postoperative positioning regimen was assessed primarily through clinical observation and patient self-report, without the use of validated quantitative assessment tools. Therefore, patient adherence, comfort, and tolerance could not be objectively quantified. Although the results are encouraging, higher-level evidence is still required for validation. It should be emphasized that this study does not negate the value of the prone position; rather, it highlights that under the guiding principle of “keeping the retinal break oriented upward,” a more feasible positioning strategy should be selected based on the break’s quadrant and pathological characteristics, so as to optimize the product of “anatomical success × patient compliance.”

## Conclusion

For patients with inferior-break rhegmatogenous retinal detachment undergoing silicone oil tamponade, adopting a side-lying position that keeps the retinal break upward is a safe, effective, and well-tolerated postoperative positioning strategy. Our findings suggest that the side-lying position may serve as an important supplement—or even a potential alternative—to the traditional prone position. This approach warrants further investigation in studies with larger sample sizes and more rigorous methodological designs, offering a new direction for postoperative management in clinical practice.

## Data Availability

The raw data supporting the conclusions of this article will be made available by the authors, without undue reservation.

## References

[1] Sultan ZN Agorogiannis EI Iannetta D Steel D Sandinha T. Rhegmatogenous retinal detachment: a review of current practice in diagnosis and management. BMJ Open Ophthalmol. (2020) 5:e000474. doi: 10.1136/bmjophth-2020-000474, 33083551 PMC7549457

[2] Kuhn F Aylward B. Rhegmatogenous retinal detachment: a reappraisal of its pathophysiology and treatment. Ophthalmic Res. (2014) 51:15–31. doi: 10.1159/000355077, 24158005

[3] Subedi S Thapa R Pradhan E Bajiyama S Sharma S Duwal S . Outcomes of microincision pars plana vitrectomy in rhegmatogenous retinal detachment. J Nepal Health Res Counc. (2023) 20:983–7. doi: 10.33314/jnhrc.v20i4.4417 37489689

[4] Pfister N Dormegny L Ballonzoli L Sauer A Speeg-Schatz C Bourcier T . Long-term microvascular remodeling and cystic changes after retinal detachment treated with silicon oil tamponade. Retina. (2023) 43:923–31. doi: 10.1097/IAE.0000000000003755, 38235973

[5] Rossi T Querzoli G Badas MG Angius F Telani S Ripandelli G. Computational fluid dynamics of intraocular silicone oil tamponade. Transl Vis Sci Technol. (2021) 10:22. doi: 10.1167/tvst.10.8.22, 34313726 PMC8322710

[6] dell'Omo R Semeraro F Guerra G Verolino M Cinelli M Montagnani S . Short-time prone posturing is well-tolerated and reduces the rate of unintentional retinal displacement in elderly patients operated on for retinal detachment. BMC Surg. (2013) 13 Suppl 2:S55. doi: 10.1186/1471-2482-13-S2-S55 24267923 PMC3850981

[7] Pan J Cheng D Feng X Zheng L Dong Y Hou Q . Effect of body position on intraocular pressure in silicone oil tamponade eyes. Retina. (2018) 38:939–44. doi: 10.1097/IAE.0000000000001633, 28376044

[8] Wang CP Huang EJ Kuo CN Lai CH. Deep vein thrombosis due to continuous prone positioning after retinal detachment surgery. Taiwan J Ophthalmol. (2016) 6:96–7. doi: 10.1016/j.tjo.2015.05.002, 29018720 PMC5602698

[9] Suzuki K Shimada Y Seno Y Mizuguchi T Tanikawa A Horiguchi M. Adherence to the face-down positioning after vitrectomy and gas tamponade: a time series analysis. BMC Res Notes. (2018) 11:142. doi: 10.1186/s13104-018-3257-1, 29463317 PMC5819221

[10] Seno Y Shimada Y Mizuguchi T Tanikawa A Horiguchi M. Compliance with the face-down positioning after vitrectomy and gas tamponade for Rhegmatogenous retinal detachments. Retina. (2015) 35:1436–40. doi: 10.1097/IAE.0000000000000479, 25748281

[11] Shiraki N Sakimoto S Sakaguchi H Nishida K Nishida K Kamei M. Vitrectomy without prone positioning for rhegmatogenous retinal detachments in eyes with inferior retinal breaks. PLoS One. (2018) 13:e0191531. doi: 10.1371/journal.pone.0191531, 29373582 PMC5786309

[12] Chen X Yan Y Hong L Zhu L. A comparison of strict face-down positioning with adjustable positioning after pars plana vitrectomy and gas tamponade for rhegmatogenous retinal detachment. Retina. (2015) 35:892–8. doi: 10.1097/IAE.0000000000000413, 25635574

[13] Cicinelli MV Benatti E Starace V Nadin F Di Nisi E Bandello F . Recurrences and macular complications after perfluorocarbon-liquid-free vitrectomy for primary rhegmatogenous retinal detachment. Ophthalmol Ther. (2023) 12:3219–32. doi: 10.1007/s40123-023-00811-z, 37775683 PMC10640444

[14] Vidne O Blum Meirovitch S Rabina G Abd Eelkader A Prat D Barequet D . Perfluorocarbon liquid vs. subretinal fluid drainage during vitrectomy for the primary repair of rhegmatogenous retinal detachment: a comparative study. Curr Eye Res. (2018) 43:1389–94. doi: 10.1080/02713683.2018.1490436, 29912572

[15] Casswell EJ Yorston D Lee E Heeren TFC Harris N Zvobgo TM . Effect of face-down positioning vs support-the-break positioning after macula-involving retinal detachment repair: the PostRD randomized clinical trial. JAMA Ophthalmol. (2020) 138:634–42. doi: 10.1001/jamaophthalmol.2020.0997, 32297923 PMC7163775

[16] Sverdlichenko I Lim M Popovic MM Pimentel MC Kertes PJ Muni RH. Postoperative positioning regimens in adults who undergo retinal detachment repair: a systematic review. Surv Ophthalmol. (2023) 68:113–25. doi: 10.1016/j.survophthal.2022.09.002, 36116526

[17] Shiragami C Fukuda K Yamaji H Morita M Shiraga F. A method to decrease the frequency of unintentional slippage after vitrectomy for rhegmatogenous retinal detachment. Retina. (2015) 35:758–63. doi: 10.1097/IAE.0000000000000383, 25341884

[18] Starr MR Obeid A Ryan EH Ryan C Ammar M Patel LG . Retinal detachment with inferior retinal breaks: primary vitrectomy versus vitrectomy with scleral buckle (PRO study report no. 9). Retina. (2021) 41:525–30. doi: 10.1097/IAE.0000000000002917, 33600131

[19] Arjmand P Felfeli T Mandelcorn ED. Combined pars Plana vitrectomy and segmental scleral buckle for rhegmatogenous retinal detachment with inferior retinal breaks. Clin Ophthalmol. (2021) 15:3515–9. doi: 10.2147/OPTH.S321371, 34434043 PMC8380623

[20] Martinez-Castillo V Boixadera A Verdugo A Garcia-Arumi J. Pars plana vitrectomy alone for the management of inferior breaks in pseudophakic retinal detachment without facedown position. Ophthalmology. (2005) 112:1222–1226.e1. doi: 10.1016/j.ophtha.2004.12.046 15939475

[21] Goto T Nakagomi T Iijima H. A comparison of the anatomic successes of primary vitrectomy for rhegmatogenous retinal detachment with superior and inferior breaks. Acta Ophthalmol. (2013) 91:552–6. doi: 10.1111/j.1755-3768.2012.02455.x, 22691313

[22] Martinez-Castillo V Verdugo A Boixadera A Garcia-Arumi J Corcostegui B. Management of inferior breaks in pseudophakic rhegmatogenous retinal detachment with pars plana vitrectomy and air. Arch Ophthalmol. (2005) 123:1078–81. doi: 10.1001/archopht.123.8.1078, 16087841

[23] Tan HS Oberstein SY Mura M Bijl HM. Air versus gas tamponade in retinal detachment surgery. Br J Ophthalmol. (2013) 97:80–2. doi: 10.1136/bjophthalmol-2012-302140, 23104901

[24] Hwang JF Chen SN Lin CJ. Treatment of inferior rhegmatogenous retinal detachment by pneumatic retinopexy technique. Retina. (2011) 31:257–61. doi: 10.1097/IAE.0b013e3181e586f9, 21052037

[25] Yee KM Sebag J. Long-term results of office-based pneumatic retinopexy using pure air. Br J Ophthalmol. (2011) 95:1728–30. doi: 10.1136/bjophthalmol-2011-300114, 21900226

[26] Narde HK Puri P Shaikh NF Agarwal D Kumar A. Vitrectomy without encircling band for rhegmatogenous retinal detachment with inferior break utilizing 3D heads up viewing system. Indian J Ophthalmol. (2021) 69:1208–12. doi: 10.4103/ijo.IJO_2028_20, 33913861 PMC8186608

[27] Alexander P Ang A Poulson A Snead MP. Scleral buckling combined with vitrectomy for the management of rhegmatogenous retinal detachment associated with inferior retinal breaks. Eye (Lond). (2008) 22:200–3. doi: 10.1038/sj.eye.6702555, 16946755

[28] Bonnar J Tan CH McCullough P Wright DM Williamson T Lois N . Scleral buckle, vitrectomy, or combined surgery for inferior break retinal detachment: systematic review and Meta-analysis. Ophthalmol Retina. (2023) 7:837–47. doi: 10.1016/j.oret.2023.05.006 37187441

[29] Rosenberg DM Ghayur HS Deonarain DM Sarohia GS Phillips MR Garg S . Supplemental scleral buckle for the management of rhegmatogenous retinal detachment by pars plana vitrectomy: a meta-analysis of randomized controlled trials. Ophthalmologica. (2022) 245:101–10. doi: 10.1159/000520220, 34731858

[30] Ghoraba HH Zaky AG Ellakwa AF. Long-term follow-up of vitrectomy, with or without 360° encircling buckle, for rhegmatogenous retinal detachment due to inferior retinal breaks. Clin Ophthalmol. (2016) 10:1145–51. doi: 10.2147/opth.s102082, 27382248 PMC4922796

[31] Mansour AM. Pneumatic retinopexy for inferior retinal breaks. Ophthalmology. (2005) 112:1771–6. doi: 10.1016/j.ophtha.2005.04.031, 16111759

[32] Chang TS Pelzek CD Nguyen RL Purohit SS Scott GR Hay D. Inverted pneumatic retinopexy: a method of treating retinal detachments associated with inferior retinal breaks. Ophthalmology. (2003) 110:589–94. doi: 10.1016/S0161-6420(02)01896-1, 12623827

[33] Sigler EJ Randolph JC Calzada JI Charles S. 25-gauge pars plana vitrectomy with medium-term postoperative perfluoro-n-octane tamponade for inferior retinal detachment. Ophthalmic Surg Lasers Imaging Retina. (2013) 44:34–40. doi: 10.3928/23258160-20121221-10, 23418732

[34] Biswas BK. Conjunctival edema and head position after prone spinal surgery. J Neurosurg Anesthesiol. (2008) 20:70–1. doi: 10.1097/ANA.0b013e3181583b23, 18157034

[35] Sun L Hymowitz M Pomeranz HD. Eye protection for patients with COVID-19 undergoing prolonged prone-position ventilation. JAMA Ophthalmol. (2021) 139:109–12. doi: 10.1001/jamaophthalmol.2020.4988, 33211075 PMC7677874

[36] Goepfert CE Ifune C Tempelhoff R. Ischemic optic neuropathy: are we any further? Curr Opin Anaesthesiol. (2010) 23:582–7. doi: 10.1097/ACO.0b013e32833e15d0, 20802327

[37] Starr MR Iezzi R. Mesenteric venous thrombosis after face-down positioning for retina detachment surgery. Ophthalmol Retina. (2018) 2:1174–5. doi: 10.1016/j.oret.2018.06.002, 31047560

[38] Abdelkader AME Abouelkheir HY. Supine positioning after vitrectomy for rhegmatogenous retinal detachments with inferior retinal breaks. Int J Retina Vitreous. (2020) 6:41. doi: 10.1186/s40942-020-00247-8, 32944286 PMC7490905

[39] Otsuka K Imai H Miki A Nakamura M. Impact of postoperative positioning on the outcome of pars plana vitrectomy with gas tamponade for primary rhegmatogenous retinal detachment: comparison between supine and prone positioning. Acta Ophthalmol. (2018) 96:e189–94. doi: 10.1111/aos.13482, 28556420

[40] Fung TH Yim TW Lois N Wright DM Liu SH Williamson T. Face-down positioning or posturing after pars plana vitrectomy for macula-involving rhegmatogenous retinal detachments. Cochrane Database Syst Rev. (2024) 2024:CD015514. doi: 10.1002/14651858.cd015514.pub2, 38488250 PMC10941635

[41] Gupta D. Rethinking surface tension and buoyancy. Arch Ophthalmol. (2011) 129:1109–10. doi: 10.1001/archophthalmol.2011.176, 21825208

[42] de Juan E Jr McCuen B Tiedeman J. Intraocular tamponade and surface tension. Surv Ophthalmol. (1985) 30:47–51. doi: 10.1016/0039-6257(85)90088-8, 4035558

[43] Gozawa M Kanamoto M Ishida S Takamura Y Iwasaki K Kimura H . Evaluation of intraocular gas using magnetic resonance imaging after pars plana vitrectomy with gas tamponade for rhegmatogenous retinal detachment. Sci Rep. (2020) 10:1521. doi: 10.1038/s41598-020-58508-3, 32001793 PMC6992615

[44] Adhi MI Adhi M Aldebasi T Hazzazi M Refka MN Omair A. Post-operative face-up or face-down positioning after silicone oil tamponade in retinal surgery: results of a retrospective study. J Pak Med Assoc. (2024) 74:1977–81. doi: 10.47391/JPMA.21480, 39548618

[45] Martinez-Castillo VJ Garcia-Arumi J Boixadera A. Pars plana vitrectomy alone for the management of pseudophakic rhegmatogenous retinal detachment with only inferior breaks. Ophthalmology. (2016) 123:1563–9. doi: 10.1016/j.ophtha.2016.03.032, 27126928

[46] Ajlan R Isenberg J Cordahi G Duval R Olivier S Rezende F. Primary rhegmatogenous retinal detachment with inferior retinal breaks postoperative prone positioning results: 1 day versus 7 days. Int J Retina Vitreous. (2017) 3:47. doi: 10.1186/s40942-017-0100-0, 29214057 PMC5713118

[47] Tornambe PE Poliner LS Grote K. Macular hole surgery without face-down positioning. Retina. (1997) 17:179–85. doi: 10.1097/00006982-199705000-00001 9196926

[48] Elborgy ES Starr MR Kotowski JG Chehade JEA Iezzi R. No face-down positioning surgery for the repair of chronic idiopathic macular holes. Retina. (2020) 40:282–9. doi: 10.1097/IAE.0000000000002396, 31972798

[49] Ye T Yu JG Liao L Liu L Xia T Yang LL. Macular hole surgery recovery with and without face-down posturing: a meta-analysis of randomized controlled trials. BMC Ophthalmol. (2019) 19:265. doi: 10.1186/s12886-019-1272-1, 31864333 PMC6925505

[50] Huang Q Cheng Y. The effectiveness of the supine position in managing inferior breaks in rhegmatogenous retinal detachment after vitrectomy with gas tamponade. Int J Gen Med. (2021) 14:1179–84. doi: 10.2147/ijgm.s306006, 33833558 PMC8021250

[51] Jonas JB Knorr HL Rank RM Budde WM. Retinal redetachment after removal of intraocular silicone oil tamponade. Br J Ophthalmol. (2001) 85:1203–7. doi: 10.1136/bjo.85.10.1203, 11567965 PMC1723734

[52] Barr CC Lai MY Lean JS Linton KL Trese M Abrams G . Postoperative intraocular pressure abnormalities in the silicone study. Ophthalmology. (1993) 100:1629–35. doi: 10.1016/s0161-6420(93)31425-9, 8233387

[53] Hutton WL Azen SP Blumenkranz MS Lai MY McCuen BW Han DP . The effects of silicone oil removal. Silicone study report 6. Arch Ophthalmol. (1994) 112:778–85. doi: 10.1001/archopht.1994.01090180076038, 8002836

[54] Scholda C Egger S Lakits A Haddad R. Silicone oil removal: results, risks and complications. Acta Ophthalmol Scand. (1997) 75:695–9. doi: 10.1111/j.1600-0420.1997.tb00633.x, 9527334

[55] Goezinne F La Heij EC Berendschot TT Liem AT Hendrikse F. Risk factors for redetachment and worse visual outcome after silicone oil removal in eyes with complicated retinal detachment. Eur J Ophthalmol. (2007) 17:627–37. doi: 10.1177/112067210701700423, 17671941

[56] Mason RH Minaker SA Marafon SB Figueiredo N Hillier RJ Muni RH. Retinal displacement following rhegmatogenous retinal detachment: a systematic review and meta-analysis. Surv Ophthalmol. (2022) 67:950–64. doi: 10.1016/j.survophthal.2022.01.002, 35007619

[57] Mahmoudzadeh R Swaminathan S Salabati M Wakabayashi T Patel D Mehta S . Retinal displacement following Rhegmatogenous retinal detachment repair. Ophthalmic Surg Lasers Imaging Retina. (2024) 55:560–6. doi: 10.3928/23258160-20240528-01, 39037356

[58] Park W Kim M Kim RY Kim JY Kwak JH Park YG . Long-term visual prognosis and characteristics of recurrent retinal detachment after silicone oil removal. PLoS One. (2023) 18:e0265162. doi: 10.1371/journal.pone.0265162, 36753472 PMC9907833

[59] Ando F. Intraocular hypertension resulting from pupillary block by silicone oil. Am J Ophthalmol. (1985) 99:87–8. doi: 10.1016/S0002-9394(14)75878-7, 3966527

[60] Al-Habsi SH Al-Abri MS. Pupillary block glaucoma due to anterior migration of nonemulsified silicone oil in a phakic patient: a case report and review of literature. Oman J Ophthalmol. (2023) 16:110–2. doi: 10.4103/ojo.ojo_92_22, 37007273 PMC10062094

[61] Nicolai M Lassandro N Franceschi A Rosati A De Turris S Pelliccioni P . Intraocular pressure rise linked to silicone oil in retinal surgery: a review. Vision (Basel). (2020) 4:32823618. doi: 10.3390/vision4030036, 32823618 PMC7558829

[62] Al-Jazzaf AM Netland PA Charles S. Incidence and management of elevated intraocular pressure after silicone oil injection. J Glaucoma. (2005) 14:40–6. doi: 10.1097/01.ijg.0000145811.62095.fa, 15650603

[63] Baskaran P Madhanagopalan VG Ramakrishnan S. A modified air-assisted silicone oil removal from the anterior chamber. Oman J Ophthalmol. (2020) 13:173–5. doi: 10.4103/ojo.OJO_196_2019, 33542613 PMC7852421

[64] Pavlidis M Scharioth G de Ortueta D Baatz H. Iridolenticular block in heavy silicone oil tamponade. Retina. (2010) 30:516–20. doi: 10.1097/IAE.0b013e3181bd2d0c, 19952994

